# Intuitive Development to Examine Collaborative IoT Supply Chain System Underlying Privacy and Security Levels and Perspective Powering through Proactive Blockchain

**DOI:** 10.3390/s20133760

**Published:** 2020-07-05

**Authors:** Aamir Shahzad, Kaiwen Zhang, Abdelouahed Gherbi

**Affiliations:** Department of Software and IT Engineering, École de Technologie Supérieure, Montréal, QC H3C 1K3, Canada; kaiwen.zhang@etsmtl.ca (K.Z.); abdelouahed.gherbi@etsmtl.ca (A.G.)

**Keywords:** Internet of Things, supply chain system, privacy and security issues, cryptography, digital signature, blockchain

## Abstract

Undoubtedly, the supply chain management (SCM) system is an important part of many organizations worldwide; over time, the technologies used to manage a supply chain ecosystem have, therefore, a great impact on businesses’ effectiveness. Among others, numerous developments have been made that targeted to have robust supply chain systems to efficiently manage the growing demands of various supplies, considering the underlying requirements and main challenges such as scalability, specifically privacy and security, of various business networks. Internet of things (IoT) comes with a solution to manage a complex, scalable supply chain system, but to provide and attain enough security during information exchange, along with keeping the privacy of its users, is the great inherent challenge of IoT. To fulfill these limitations, this study designs and models a scaled IoT-based supply chain (IoT-SC) system, comprising several operations and participants, and deploys mechanisms to leverage the security, mainly confidentially, integrity, authentication (CIA), and a digital signature scheme to leverage potentially secured non-repudiation security service for the worst-case scenario, and to leverage privacy to keep users sensitive personal and location information protected against adversarial entities to the IoT-SC system. Indeed, a scaled IoT-SC system certainly opens new challenges to manage privacy and security while communicating. Therefore, in the IoT-SC system, each transaction writes from edge computing nodes to the IoT-SC controller is thoroughly examined to ensure the proposed solutions in bi-directional communication, and their robustness against adversarial behaviors. Future research works, employing blockchain and its integrations, are detailed as paces to accelerate the privacy and security of the IoT-SC system, for example, migrating IoT-centric computing to an immutable, decentralized platform.

## 1. Introduction

Internet of Things (IoT) is a centralized platform that provides reliable connectivity among objects, such as devices, sensors, machines, actuators, or things that can exchange information over the internet. IoT, together with its number of applications, offers several opportunities to manage and monitor the overall information exchange between hundreds of thousands of connected devices [1,2]. Among others, the available analytical features of the IoT empower useful actions on carried information, therefore are beneficial for several businesses to make highly intuitive decisions and actions accordingly. However, generally speaking, IoT is still a growing technology, in terms to manage an extensively large network and information exchange between a large number of connected devices, therefore leads the great challenges of, for example, scalability, privacy, and security issues [2,3]. Among other challenges [3,4,5], privacy and security are the most common unaddressed issues of IoT, which, therefore, have harmful effects on the system or IoT network performance. Like in traditional networks, several vulnerabilities and threats have been examined in the IoT communication system, including denial-of-service (DoS) attack and distributed denial-of-service (DDoS) attack, which caused a great disruption between devices data exchanging [4]. Thus, a weak, insecure, IoT platform leads to various potential cyber-attacks. As mentioned, scalability is considering a major challenge for IoT, scalability issues arising due to the massive congestion, for example, the growing number of devices connectivity; therefore, scalability can cause further issues of privacy, such as users/objects information protection, and security, such as authentication and authorization, which therefore are challenging tasks to be managed by the IoT centralized system. The cost will be necessarily increasing if IoT organizations start deploying an expensive, high computing server to manage and control massive traffic congestion, and to maintain privacy and security during information exchange. Conventionally, scalability is an inherent property of IoT; however, a scaled-IoT platform can cause several vulnerabilities with cyber-criminals, especially issues of privacy and security [5,6]. 

Supply chain management (SCM) systems are the most crucial parts of industries and organizations; a well-defined, effective SCM can directly influence operations, productions, and profitability of organizations [7,8,9]. As the time past, to fulfill a massively growing demand of the supply chain over the world, industries, organizations, or firms, have been adopting the advanced technologies, such as radio frequency identification (RFID) and sensing solutions, artificial intelligence (AI), and cloud computing to manage the extensively large and complex operations of the modern supply chain ecosystem [9,10,11,12,13]. Thus, gathering and managing large data in the complex and large-scale supply chain, in which entities and operations involved are distributed over various locations, undoubtedly is a big challenge. However, employing AI, machine learning, and big data in the supply chain can be solutions to address challenges of data collection, analyses, and processing [10,14,15]. Through the integration of IoT, supply chain systems are much smarter than ever, as IoT smart sensing technology and devices connectivity enable the supply chain systems to generate and collect massive data and to monitor and control the overall supply chain ecosystem, therefore leveraging great transparency, tracking, and central security features [16,17]. In the past, a number of research studies have been conducted to use IoT solutions to manage the supply chain operations, workflow, and processing; the conducted research works are good enough to support the industrial productions and deliveries, in short, the ultimate target is to generate profit; however, there are limited concentrations paid to the main challenges: privacy, security, and scalability, associated to the supply chain or IoT integrated supply chain either. IoT distributive connectivity and centralized paradigms are not very supportive to provide and ensure system privacy and security of a large-scale supply chain system and its operations [17,18]. For example, IoT smart sensors and devices usually are low cost and have limited resources (e.g., limited energy consumption), design, and manufacture from distinct manufacturers whose main intensions are not to examine and embed the features of autonomous security and privacy for IoT open connectivity. IoT uses entirely different smart sensors, protocols, standards, and devices, from different manufacturers and firms, therefore, to attain performances of security and privacy are challenges for IoT platforms [19,20,21]. All these gaps, such as security and privacy, can be bridged with various solutions: encryption and cryptography, digital signature schemes, anonymous identity, and location hiding solutions [22,23,24,25]. IoT, together with its advanced applications and analytical tools, enables an efficient, accurate, granular, and flexible SCM system to manage all its operations and workflows [9,16]. In short, IoT is an ultimate solution to the supply chain, a system that is fully operational and robust as according to modern demands of the current era; therefore, employing IoT solutions to SCM, organizations can save much cost that they are spending for granular and analytical data provision and are able to get direct access to their data with more accuracy and efficiency.

As mentioned in [9,18], several organizations have started employing the Internet of Things (IoT) to manage and monitor the whole supply chain ecosystem, for example, by tracking and monitoring assets and supplies in real time. With IoT enabled featuring, including analytical processing, supply chain systems become more efficient, however, there have been limited intentions paid to underlying main issues of privacy and security [4,19]. On the other side, IoT inherently lacks to provide significant enough information security and to keep the privacy among its numerous interconnecting objects and components, these issues may be arising more as consequences of IoT scalability and interoperability [4,26]. IoT is a central platform to manage its millions of connected objects, mainly under-designed distinct manufactures and suppliers, employing a variety of software applications to drive processing in the IoT platform. Therefore, there are numerous ways in which IoT can be affected by various threats, vulnerabilities, and cyberattacks [6,7]. The integration of IoT into the supply chain, therefore, comes with a new source of vulnerabilities and attacks, which therefore gives insight to have more robust and secure communication systems [4,5]. For example, the supply chain system is not a central computing system, its entities and operations are distributed across several locations, therefore taxonomy of potential risks is unknown and high in the supply chain ecosystem; adopting new technologies and applications, even integration of IoT, the small and large organizations should do a risk analysis before to update the existing infrastructures; IoT integrations always require system updates, e.g., hardware/software updates, to keep managing its network effectiveness time-to-time. 

In this study, we first thoroughly examined the underlying potential issues, mainly concerned with privacy and security, of IoT and supply chain systems in terms of data collection, generation, and manipulation. Then, we implemented an IoT-based supply chain (IoT-SC) system, a model and design to provide IoT integration to end-to-end supply chain systems and used the Cloud SQL database to record each transaction. From the literature, we explicitly conclude that confidentiality, authentication, integrity, and non-repudiation are important services which develop employing cryptography and digital signature schemes, i.e., advanced encryption standard (AES), SHA-256, and RSA public-key cryptography algorithm, could conquer the main security challenges of IoT-SC; and we also conclude that identity and location are main privacy challenges in IoT-SC, which could resolve through using of cryptography and digital signature, anonymity and pseudonym, and location-based services, respectively. Later, formal proofs and conducted experimental results entirely examine the effectiveness of IoT-SC against the given security and privacy challenges.

The rest of the paper is organized as follows. Section 2 conducts a detailed literature review on existing studies: supply chain management, IoT and supply chain management, and security and privacy challenges and developments. Section 3 details a background study on IoT and the supply chain system. Section 4 demonstrates a privacy and security analysis. Section 5 designs and models an IoT-SC system, conducts a formal statement (or proofs) to examine privacy and security services, and highlights some main limitations and enhancements. Section 6 details the results and makes discussions to evaluate the effectiveness of the proposed study. The overall study concludes in Section 7, and Section 8 provides some interesting future directions using blockchain technology.

## 2. Literature Survey 

Privacy and security are the fundamental issues for a large-scale IoT system, as IoT objects are largely distributed in various locations, therefore IoT centric-computing platforms are inherently not very efficient to provide and manage these issues, e.g., while communicating to millions of objects [4,6]. In IoT smart home applications, sensors and devices are networked to generate data that is important to manipulate further to take actions; however, with information exchange between devices there are certainly several security risks: security and privacy issues [2]. “GHOST (Safeguarding home IoT environments with personalized real-time risk control) project, or European research project GHOST”, was a large IoT project which mainly targeted to develop a reference architecture to address the cybersecurity issues to IoT networks [27].

Considering the issues, such as information complexity and availability, in supply chain management, and to make the SCM smarter in information manipulation, a secure and effective SCM system is built using the Internet of Things (IoT) [4]. Through IoT connectivity, information is traced at each stage of SCM applying RFID technology, e.g., an online system or web application is used that is accessed by both supplier and manager, and product scanning using embedded RFID tags is done to ensure traceability at each stage of the supply chain. Further, to examine the security in the SCM system, the analytics and evaluation methods, such as neutrosophic Decision Making Trial and Evaluation Laboratory (N-DEMATEL), are used [4,28]. Supply chain management is considered a complex system compared to other ICT systems, as it is comprising several entities and operations in the supply chain [9]. Further, the complex structure of SCM and its interconnectivity through IoT, the possibility of threats, and risks, therefore, will be large [17]. For that, potential security risks and challenges are examined for IoT-based supply chain systems [4,17]. Dos Santos and Canedo [29] developed an IoT system that employs RFID tags embedded to objects to read information, cloud computing (i.e., Microsoft Azure) technology and microservices, and independent IoT services, to manage the scalable system and a large dataset generated from ultra-high frequency (UHF) RFID tags. The main challenges, such as collision, security, privacy, associated with RFID technology are examined in [30]. IoT technology has great importance to boost the supply chain and further to make significant decisions on information carried from nodes, e.g., RFID tags and sensors [31]. A study on logistic management, with the integration of IoT and cloud computing services, e.g., Software-as-a-Service (SaaS), was conducted as a practical implementation to build a manufacturing system and to carry its operations [32]. In [33], IoT uses a virtualization platform to facilitate the operations of the food supply chain, for that, a proof of concept implementation, i.e., considering a use case of a fish supply chain, is made to demonstrate the effects of autonomous operations, decision and learning supports of an IoT network. IoT virtualization, therefore, has better support to monitor perishable products during the supply chain remotely.

Increasing in the demands of supply in industries, supply chain systems have been evolving with new emerging technologies [6,9], for example, blockchain is one among them [34]. Litke et.al. [35] conducted a study to examine the blockchain and its applications, such as security and privacy through consensus mechanism, scalability, and other performances like transparency and traceability, which have importance to bridge the gaps in the SCM system, and to achieve better productions and profitability. Feng Tian [36] used a term called “HACCP (Hazard Analysis and Critical Control Points)” and selected a use case to provide transparency in the food supply chain system, considering overall system entities, employing IoT and blockchain technologies. IoT and blockchain can support numerous features to the food supply chain to achieve better transparency, traceability, reliability, privacy, and security [30]. IoT enabled technical features and connectivity using smart sensors, RFID and GPS provide a reliable platform to collect information having transparency in real time, and the information continuously stores into BigChainDB, which has similar characteristics of the distributed database, as well conventional blockchain. Food products are traced through embedded RFID tags, and the participants can check, add, and write information, by keeping their authorized identities, to the supply chain system [9,36]. Existing warehouse management systems are not much efficient and somehow inappropriate to conquer the increasing demands of customers and to provide a sound system that can reduce managing cost spend on large and complex inventory in warehouses [32,33]. In the past, several technological solutions have been developed for warehouse management, however, most of the solutions are not effective in management, lacking tracking mechanism, and undoubtedly require enough manpower, therefore degrading system performances [37,38]. IoT can be a better solution for warehouse management, which enables massive connectivity of objects, for example, connectivity to collect and exchange information of products or objects using RFID and sensing technologies [38,39]. By doing so, the warehouse management system can increase its visibility of products in real time, and enhance the processing speed through tracks, which therefore increases the system performance in more efficient ways [38]. Blockchain provides great transparency for inventory management and counterfeiting [40]. For IoT-based warehouse inventory management and the supply chain system [41,42], the issues of privacy and security can be solved by employing cryptography and information hiding mechanisms [5,6,22,23,24,25]. For IoT, cryptography solutions [40,43], such as symmetric and asymmetric solutions, are the best solutions to provide and gain security or confidentiality in unsecured transmission. Both solutions used complex encryption and decryption operations, having different key pairs and sizes, to secure transmission against vulnerabilities and attacks [5,40].

IoT adoption in various systems and applications has been increasing rapidly, a report stated that IoT objects connectivity will reach 21 billion by the year 2025 [44]. IBM has built a generic IoT platform, incorporated with other evolutionary technologies like blockchain and artificial intelligence, this IoT platform can also be used with other technologies such as deep learning, future security designs, edge/fog computing, etc. [45,46].

## 3. Background

### 3.1. Internet of Things

Over the years, the Internet of Things (IoT) has been getting great popularity due to its massive advancements in several sectors such as supply chain management, manufacturing, and industries. IoT is an emerging technological concept that enables connectivity for everything, things such as IoT devices and smart sensors, which can sense information, for example, from the physical environment, and can exchange information with other objects or to the IoT server. The collected information will further manipulate and analyze therefore to make useful decisions to provide end-users services and to take actions to manage IoT systems. Furthermore, central cloud technology is used as a storage management system in the IoT platform [47]. In short, IoT is a growing advanced technology of this era, which has great technological potential and applications, e.g., online smart web applications and services, to provide connectivity to the physical world and further to process and manage information from millions of networked objects.

IoT is mainly composed of five layers [47,48]: “(1) Perception or physical layer, (2) Network or communication layer, (3) Middleware or processing layer, (4) Application layer, and (5) Business layer”. In some situations. IoT only uses a 3-layer communication model, which contains: “(1) Perception layer, (2) Network layer, and (3) Middleware or processing layer”. The main uses of IoT are to provide reliable interconnectivity between larger objects, e.g., devices and smart sensors, and to support high-level computation and analytics for further processing and examining data for end-user perspectives. The following are the details of the IoT defined five layers model including each layer services.

- Perception Layer: Like the traditional open system interconnection (OSI) model, the IoT communication model also uses a bottom-up approach. In the IoT communication model, the perception layer or physical/device layer is the most bottom layer and provides connectivity to a number of devices, smart sensors, machines, actuators, other equipment able to collect and exchange information over the Internet. These devices and sensors collect information from the physical world, or other appropriate sources, and process collected information to the network layer, along with their unique identifications or addresses. The addressing scheme is mainly internet protocol addressing scheme: IPv4 and IPv6.
- Network Layer: The network layer, or transmission layer, collects data, for example, fixed or variable length packets, from the perception layer and further processes to the upper layer or middleware layer. The middleware layer is considered a processing layer in the IoT communication model. The connectivity is done through networked routers and access points, employing communication media such as wired and wireless. For communication and data exchange, a number of protocols and standards are used by IoT, such as Bluetooth, Wi-Fi, RFID, NFC (near field communication), LTE (Long-Term Evolution), cellular protocols, IEEE (Institute of Electrical and Electronics Engineers) communication standards, and others.
- Middleware Layer: This is one of the main layers in the IoT model, supporting a massive number of applications, services, and storage systems or cloud storage systems. Indeed, IoT is a network of millions of objects, a specific number of objects are networked to perform specific measurements, therefore this layer is highly responsible to perform a large number of tasks: services or end-user services, data storage, data manipulation, analytical decisions on information or dataset, etc.
- Application Layer: The end-user can interact with its applications and services through the application layer, or sometimes called the user application layer. This layer enables data management, for example, in different formats, collecting data from the middleware layer, and responsible to provide various services to end-user requests.
- Business Layer: This layer is called a master layer or controller layer, which is responsible to manage the entire IoT network, including various types of business use cases, services, and innovative applications. Through this layer, organizations, and enterprises can build and deploy their proposed business strategical models and further to collect analytical measurements based on their deployed models.

### 3.2. Supply Chain System

Supply chain systems are very essential systems for numerous industries and organizations, mainly the various businesses reproduction and profitability directly depending on the effectiveness of the supply chain system used [47,48]. A supply chain system encompassing several users/entities and operations; however, a number of participating entities and their performing operations depend on the type and size of organizational infrastructure. For example, in the food supply chain system, there are also a number of entities and operations involved, depending on the type of food products: perishable and non-perishable food products. Here are some common entities and their operations used in the food supply chain system, which are: (1) Farmer, responsible to manage raw material; (2) Manufacture, responsible for packing raw material to standard size boxes; (3) Warehouse (or distribution center), responsible to perform inventory storage and distributions; (4) Retailer, responsible to collect, manage products, and to deal the customers; (5) Transporter, responsible to collect the products and provide deliveries to various specified locations; (6) Customers, these are actual entities or end-users, may or not be parts of the supply chain, who purchase and use products [49].

In the supply chain ecosystem, it is important to demonstrate each participant’s participation and access rights; in most cases, there are fewer intensions paid on a farmer and final customer entities, however, their roles are important to conduct a robust end-to-end supply chain system. At the farmer’s side, we need to know how the food products were actual growth and processed. For example, here are some main queries that need some feedback: (1) Products (or items) were grown in open-air agriculture fields or greenhouses or usage of other similar resources? (2) Were there any environmental effects during growth? (3) What type of materials were used during growth, like pure natural fertilizer or artificial fertilizer made by some chemicals? (4) Was quality assurance of all materials used in growth certified from ISO standards or some other standard organization? (5) Did some quality assurance policies satisfy the total quantity of raw materials before delivery to manufacturing? These are some important questions that a robust supply chain system must keep in consideration; these essential considerations will be very helpful while tracing any product specifically from farmer to end. IoT can be a fine-grain solution to resolve all these mentioned issues or to give feedback to all mentioned queries [50]. We assume that this will be an interesting future use case of IoT and the supply chain. For the customer end, it is not important to provide overall actual information to the customers; customers are always interested to know: the delivery date of raw materials to the manufacturer, manufacturer manufacturing date and expiry date of products, and product ingredients and its labeling to the products. However, in this study, we do not have much detail of overall internal processing and operations involved in the supply chain, because our main objectives are to implement and examine the proposed solutions, such as privacy, security, for IoT-SC system.

## 4. Security and Privacy Analysis

IoT is a scalable platform, therefore scalability is considered one of the major challenges that still is required to settle down to stabilize the effectiveness and robustness in the IoT massively distributed platform. IoT provides a centric-computing paradigm to collect, monitor, and control information from its connected objects; the connectivity is distributive as the objects are connected across various locations, having distinct requirements of devices configurations, protocols, and communication standards. Therefore, because of these requirements and communication challenges, the IoT controller is usually unaware of what is happening at remote sites. For that, enormous applications and services installed as an add-in to IoT devices, e.g., edge/fog nodes, of which users are to supervisor some of the computation locally, without the supervision of the IoT controller [3,5]. Scaling the IoT networks can therefore equally increase security and as well as privacy issues [6,40]. For the scalable IoT system, the main security issues of confidentiality, integrity, and authentication, or CIA, are raised when IoT communicates to its objects having distinct configuration requirements, for example, large interoperability issues; on the other side, privacy issues mainly rising when there are no proper identifications of devices during communication, for example: one device is authorized to exchange its private information to another device, by keeping target device valid identity; the originator device can communicate to another, by keeping its identity anonymous or hidden.

For a large scale and distributive system, for example, the food supply chain management (SCM) system, where most of the participating entities are unknown to each other, but are known to the main controller. Thus, deploying IoT integration to the food supply chain can get great visibility, monitoring, controlling, and tracking in the entire supply chain ecosystem; however, IoT integration is not much effective to provide enough security and privacy measurements during information exchange in the supply chain systems. The SCM system is a concept of a large and complex system composed of several entities and operations, therefore, IoT integration moves the SCM system in a more scalable and complex system [26]. So, we say that the Internet of Things (IoT) and supply chain systems both are scalable systems, composed of a number of devices, operations, applications, and services to manage the overall system performing tasks [9,36]. As time past, SCM systems were upgraded by employing new technologies such as RFID, sensing, and analytical processing; therefore, it is interesting and demanding to develop an SCM system, with end-to-end connectivity, employing IoT technology [30,33]. IoT provides several new features of collecting and examining information that surely improves SCM system performance. However, among others, integration of IoT with SCM, this integrated system may suffer from various possibilities of privacy and security issues; these issues can occur internally as parts of supply chain operations and occur by considering the specific cases of IoT open interconnectivity and information exchanging features to the supply chain. Therefore, it is important for the supply chain system to examine all possible issues of security and privacy before deploying new technologies, as SCM systems are already suffering from various challenges of traditional networks and communications [4]. For example, in the case of IoT adaptability in SCM.

In this study, we examined that the IoT-based IoT supply chain (IoT-SC) system should require all essential security services such as authentication, integrity, confidentiality, and non-repudiation to combat the vulnerabilities and attacks, even these are important aspects that every internet-based system should adopt to attain its performance, for example, to ensure communication against unauthorized access and tampering. Another main issue we examined as part of the proposed study are privacy issues to the IoT-SC system. Privacy is a common challenge individually to IoT and SCM, such as privacy of user identity and location. Therefore, we targeted to examine and test these security and privacy challenges of the IoT-SC system. Considering this extensive scenario, there is essentially a requirement to have robust security and privacy models for the IoT-based supply chain (IoT-SC) system, where each node can communicate to the IoT-SC controller, or other nodes in the IoT-SC system, with proven of information security, i.e., CIA, and privacy of every node sensitive information, user/node identity, and location, are the common privacy issues in IoT-SC system.

## 5. System Design and Modeling

**Figure 1:**
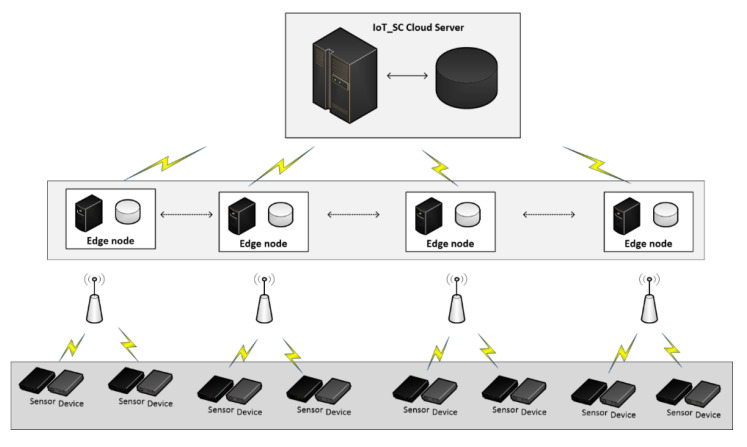
IoT-based supply chain system.

This study considers n number of nodes, these are IoT objects such as devices or smart sensors, represented by a set $S_{1}$, $S_{1}=\left\{{\left(node\right)}_{1},{\left(node\right)}_{2},\ldots \ldots ,\;{\left(node\right)}_{n-1},{\left(node\right)}_{n}\right\}$, where n is a fixed value depending on the size of IoT-SC system, i.e., $S_{1}=\left\{{\left(node\right)}_{n,\;n=n-1}\right\}$. Number of nodes in a set $S_{1}$ are not autonomous nodes, these are managed and controlled by a number of edge nodes represented by a set $S_{2}$, $S_{2}=\left\{{\left(edge\right)}_{1},{\left(edge\right)}_{2},\ldots \ldots ,{\left(edge\right)}_{e-1},{\left(edge\right)}_{e}\right\}$, where e is also a fixed value depending on the size of the IoT-SC system, i.e., $S_{2}=\left\{\left(\mathrm{edge}{)}_{e,e=e-1}\right)\right\}$. Meaning that, the number of edge nodes in a set $S_{2}$ are directly proportional to entities, such as farmer, manufacture, warehouse, etc., associated to the IoT-SC system. These entities may belong to, or represent, one or more organizations that participate in the IoT-SC system. Edge nodes are partially autonomous nodes, which means that these nodes can provide some services, such as temporary data storage, error detection, and reporting, and nodes status check, and are also connected to the IoT-SC system. Edge nodes are limited in resources, such as computing power and storage, therefore, these nodes are configured and networked to process the collected information after a period of time to the IoT-SC controller CS. CS is superior in the IoT-SC system. Edge nodes perform a number of read $R_{\left(r\right)}$ and write $W_{\left(w\right)}$ transactions, $T_{\left(R,W\right)}$, $T_{\left(R,W\right)}=\left\{\left(T_{\left(R_{\left(r\right)},\;W_{\left(w\right)}\right)}{)}_{t,t=t-1}\right)\right\}$, representing a set $S_{3}$, i.e., $S_{3}=T_{\left(R,W\right)}=\left\{\left(T_{\left(R_{\left(r\right)},\;W_{\left(w\right)}\right)}{)}_{t,t=t-1}\right)\right\}$, where r,w∈t. Number of transactions $T_{\left(R,W\right)}$ can be carried out by each edge node in a set $S_{2}$, each edge node can read transactions $T_{R_{\left(r\right)}}$ from nodes in a set $S_{1}$, record all carried transactions, i.e., transactions $T_{W_{\left(w\right)}}$, into local temporary storage, and after a time period, these numbers of transactions $T_{W_{\left(w\right)}}$ are written to CS. However, edge nodes are only allowed to write transactions to CS, and can receive instructions from CS, but not allowed to read any information stored onto CS [51]. As per defined permission access rules, authorized entities can get and trace information through accessing the IoT-SC controller via some web applications. Web application and development is not under the scope of this study, as we can get and trace information through accessing locally to the IoT-SC controller. We assume that identity I and exact location L or GPS coordinates, such as latitude and longitude, of nodes in a set $S_{1}$ and edge nodes in a set $S_{2}$ are known and recorded onto CS. As per communication rules, CS allocates a specific number of nodes to specific edge nodes, by doing so, an edge node, e.g., ${\left(edge\right)}_{1}$, can keeps information on those specific nodes identities and exact locations; in other words, CS writes information to a specific edge node. For example, if we set a value of n to 10 in a set $S_{1}$, to allocate 10 nodes, each node has a unique identity I and location L, i.e., $S_{4}={\left(I,\;L\right)}_{S_{1}}=\left(I_{S_{1}},\;L_{S_{1}}\right)$, to ${\left(edge\right)}_{1},\;e=1\in \;S_{2}$, thus $\sum_{n=1}^{10}{\left(node\right)}_{n}$ number of nodes are connected to ${\left(edge\right)}_{1}$. Number of nodes identities and locations are composed in a set $S_{4}$, and a set $S_{5}$ representing the number of edge nodes identities and locations, which are known to CS. Even, in the situation of edge to edge communication, each edge node identity and location representing in $S_{5}$, i.e., $S_{5}={\left(I,\;L\right)}_{S_{2}}$, are not known to other edge nodes in a set $S_{2}$, because of privacy issues, each edge has to verify a valid identity and location of another edge node through the IoT-SC controller before initiating any transaction. For example, a receiving edge node can verify a valid identity of the originator through CS. Figure 1 demonstrates a detailed network architecture of the IoT-SC system.

**Figure 2:**
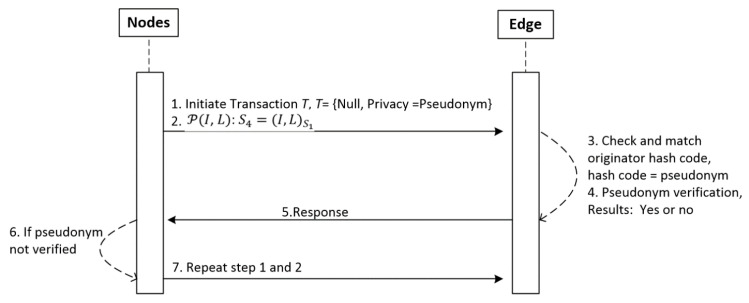
Privacy processing: numbers of steps are used to process the privacy statement, considering communication case 1: nodes to CS communication; therefore, in similar manners, we can process the privacy statement for case 2, case 3, and case 4.

**Figure 3:**
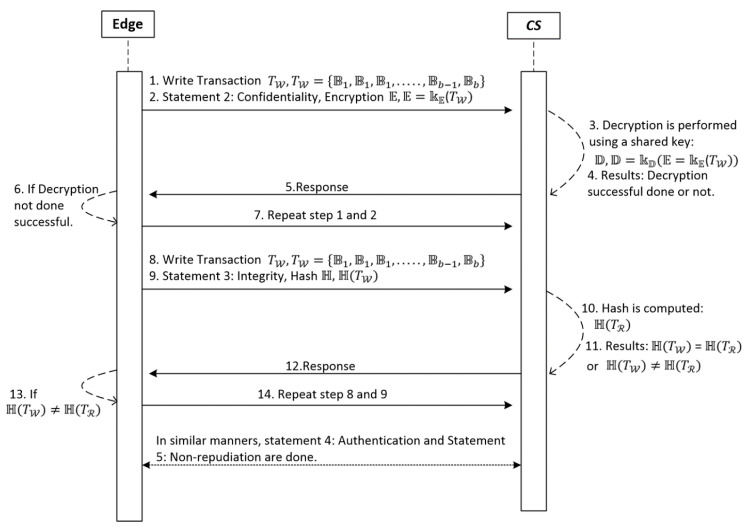
Security processing: numbers of steps are used to process the security statements. Statement 2: Confidentiality and Statement 3: Integrity, considering communication case 5: edge to CS communication and case 6: edge to edge communication, as case 5 and case 6 are logically similar in communication; therefore, in similar manners, we can process the security statements 4 and 5, considering communication case 5 and case 6.

To demonstrate the robustness of the IoT-SC system, considering potential cases of privacy and security, we set 6 main statements. Statement 1 is a privacy statement, detailed in Figure 2, which proves and examines the privacy of the IoT-SC system using communication cases: case 1: nodes to edge communication, case 2: nodes to CS communication, case 3: edge to edge communication, and case 4: edge to CS communication. Statement 2 to 5 are the security statements, detailed in Figure 3, which in turn prove and examine the security of the IoT-SC system using communication cases: case 5: edge to CS communication, and case 6: edge to edge communication. For each case, we create a potential scenario where the adversary has some possibilities to interfere directly/indirectly into the communication and therefore violates the security and privacy of the IoT-SC system. Table 1 depicts some main terminologies used in the proposed system design and modeling.

### 5.1. Statement 1: IoT-BC Privacy

Privacy P can be achieved by hiding sensitive information, a unique identity I and location L, so each node can communicate as a pseudonym, employing cryptography capabilities. For the certain defined communication cases, probability P of P(I, L), i.e., $P{\left(P\right)}_{p}$, of each successful attempt, p is greater than probability P of adversary A, $P\left(A_{p}\right)$; in other words, the $P{\left(A_{P}\right)}_{p}$ for each successful attempt, p is approximate zero, $P{\left(A_{P}\right)}_{p}=0$.

In the IoT-SC system, keeping a verifiable privacy P, such as valid identity I and location L, of each node in sets $S_{1}$ and $S_{2}$ is the main concern, which means that each node’s privacy, P(I, L), should be protected for every transaction in a set $S_{3}$, $S_{3}=T_{\left(R,W\right)}=\left\{{\left(T_{\left(R_{\left(r\right)},\;W_{\left(w\right)}\right)}\right)}_{t,t=t-1}\right\}$, where r,w∈t. We used cryptography hashing the SHA-256 algorithm to compute a hash code H on P=(I, L), and record on the IoT-SC controller. By doing so, a unique hash code H is used as a pseudonym identity for each node in the IoT-SC system. In reality, as we also model our IoT-SC system, if we have n of nodes in a $S_{1}=\left\{{\left(node\right)}_{n,\;n=n-1}\right\}$, and have e number of edge nodes $S_{2}=\left\{\left(\mathrm{edge}{)}_{e,\;e=e-1}\right)\right\}$, therefore, it is not possible to compute a unique hash code for each node/edge node of $S_{1}$ and $S_{2}$, and record on the IoT-SC controller. A set $S_{4}$, $S_{4}={\left(I,\;L\right)}_{S_{1}}$, representing each node’s identity I and location L of $S_{1}$, $S_{5}={\left(I,\;L\right)}_{S_{e}}$ representing each edge node’s identity I and location L of $S_{2}$, and CS identity I and location L are uniform. Therefore, we can distinguish each node and edge unique identity I and location L by sets $S_{4}$ and $S_{5}$. For a given problem, to compute and record a unique hash code, two possibilities exist:

- In the first situation, the IoT-SC system models for some fixed number of nodes and edge nodes, such that values of n and e are fixed and known to CS, then the system is much efficient to compute and record a unique hash code for a given value of n and e. Therefore, a system can examine the privacy of each node and edge node by verifying hash codes recorded on CS. If an adversary A exists to steal the personal information, P(I, L), of any node or edge node, and $P\left(A_{p}\right)$ is very low or is approximately zero, therefore, an adversary A may successful steal the hash code but not the actual information behind the hash code.
- In the second situation, IoT-SC models as a partially autonomous system. This means that there are some fixed number of nodes and edge nodes, such that n and e are some fixed values, which networked, and are known to CS. In addition, their hashes are also recorded onto CS. If there are some external nodes or partially autonomous nodes that wish to communicate into the IoT-SC system. These partially autonomous nodes can grant permissions, after recording of hashes, from CS. In a situation, an adversary A, or any unknown node, acts to steal the personal information, P(I, L), of another valid node or edge node, and $P{\left(A_{P}\right)}_{p}$ is very low or is zero, adversary A may successfully steal the hash code but not the actual information behind the hash code. However, in a situation, if a fully autonomous node wishes to initiate communication, and act as an adversary time-to-time to steal sensitive information from other valid nodes, $P{\left(A_{P}\right)}_{p}$ of stealing information is still very low or is approximately zero, as adversary A cannot steal actual information behind the hash code.

### 5.2. Statement 2: IoT-BC Confidentiality

A strong confidentiality C can be attained for each transaction $T_{\left(W,R\right)}$ in a set $S_{3}$, if there exists a unique shared key $k_{\left(E,D\right)}$ perform encryption E and decryption D, and satisfies the requirements of the communication system. If there exists an adversary A and the probability P of information leakage by $A_{C}$ is approximately zero, $P\left(A_{C}\right)$ = 0 for each $T_{\left(W,R\right)}$.

To demonstrate and examine the confidentiality C of each transaction $T_{\left(R,W\right)}$, $T_{\left(W,R\right)}:$ $T_{W},T_{R}$. Two main communication cases exist: case 5: edge to CS communication, and case 6: edge to edge communication. For both cases, we assume that each transaction $T_{W}$ is a payload composed of random bits, and we use the symmetric algorithm, i.e., advanced encryption standard (AES), to perform encryption E on $T_{W}$ and decryption D when the information will be read as transaction $T_{R}$, employing a unique key $k_{\left(E,D\right)}$ $k_{E},k_{D}$. For each $T_{\left(W,R\right)}$ in a set $S_{3}$, the key is always a unique 256 bits key, generated through the randomization process, and shared securely at both sides of the transmission. Therefore, the encryption E and decryption D functions, employing a common unique 256 bits key, $k_{\left(E,D\right)}$, are given as $E=k_{E}\left(T_{W}\right)$ and $D=D\left(E\right)=k_{D}(E=k_{E}$ ($T_{W}))=T_{W}$.

E and D simply demonstrate encryption and decryption functions, employing the symmetric algorithm, without detailing the complexities of encryption/decryption operations. Encryption E is performed on $T_{W}$ is a payload that transmitted from edge nodes (any) in a set $S_{2}$, and decryption D is performed when information read as transaction $T_{R}$ by edge nodes (any) in a set $S_{2}$ or by CS, employing a unique shared $k_{\left(E,D\right)}$.

Suppose that, an adversary A may have enough computing power, and has access to some tools or mechanisms to launch confidentiality attacks, such as eavesdropping and sniffing [51]. An adversary A tries to launch a number of confidentiality attacks ${\left(A_{C}\right)}_{c}$, where c represents the possible number of confidentiality attacks, to interfere in transmission continuously and therefore to leak information, or transaction $T_{W}$, composed of random bits. In our case, a unique shared key $k_{\left(E,D\right)}$ is important; as mentioned, for case 5 and 6, $k_{\left(E,D\right)}$ is shared securely to perform encryption E and decryption D before to initialize any transaction $T_{W}$. However, there may some probability P exist that an adversary A can guess the keys to perform decryption, as unauthorize entity to leak transaction $T_{W}$, before an actual node read transaction $T_{R}$, followed by cases 5 and 6. Thus, we can examine the effectiveness of our security approach and the power of the adversary $A_{C}$, by estimating $P{\left(A_{C}\right)}_{c}$ of success and fail depending on the value of c. However, we do not care how many times an adversary A launched confidentiality attacks ${\left(A_{C}\right)}_{c}$ and is successful, we do care how many times an adversary A succeeded to leak all or some of bits from each transaction $T_{W}.$ For example, if $P{\left(A_{C}\right)}_{c}\left(success\right)$ is greater than limits of the lower bound, or $P\left(A_{C}\right)$ = 0 for each $T_{\left(R,W\right)}$, thus we may conclude that security solutions attained enough security in the IoT-SC system.

### 5.3. Statement 3: IoT-BC Integrity

H is a fixed length, unchangeable hash value and computes on each transaction $T_{\left(W\;,\;R\right)}$ in a set $S_{3}$, to verify the number of actual bits, and alteration bits, such that $T_{W\;}$= $T_{R}$. A condition $T_{W\;}$= $T_{R}$ is true, if the computed individual hash value H of $T_{W\;}$ and $T_{W}$ is the same, i.e., $H\left(T_{W}\right)$ = $H(T_{R})$, and the probability P of information alteration by an adversary $A_{I}$ is approximately zero, $P\left(A_{I}\right)$ == 0 for each $T_{\left(W\;,\;R\right)}$.

Information integrity is an important security service for communication systems, for example, the IoT-SC system. For the IoT-SC system, to verify information or $T_{\left(W\;,\;R\right)}$, considering both defined cases: edge to edge and edge to CS, is an important security challenge to overcome. Meaning that, by deploying the integrity mechanism, the IoT-SC system ensures that each transaction $T_{W\;}$, composed of random bits, has not been altered during transmission, and will be verified, i.e., $T_{W\;}$= $T_{R}$. To achieve that, we use cryptography hashing H to compute an unchangeable hash value for each $T_{\left(W\;,\;R\right)}$, considering case 5 and case 6. For example, let us assume that $T_{W}$ is the written transaction composed of random bits pattern $B_{b}$, i.e., $T_{W}=\left\{B_{1},B_{1},\;B_{1},\ldots \ldots ,\;B_{b-1},\;B_{b}\right\}$, where b is some fixed value, and H($T_{W})$ is a hash of $T_{W}$, periodically transmits, and received. Meaning that, H($T_{W})$ will be unique for each written transaction $T_{W}$. Thus, for each transaction $T_{\left(W\;,\;R\right)}$, H($T_{W})$ is the computed hash of $T_{W}$, H($T_{R})$ is the computed hash of $T_{R}$, and both values are computed employing SHA-256. Therefore, we can say that, $H_{W}$= H($T_{W})$ and $H_{R}$= H($T_{R})$. Similarly, $H_{W}=H_{R}$, this condition will be true if the contents of $T_{W\;}$ have not been changed during communications, case 5 and case 6, and verified by computing $H_{R}$. In situations, when an adversary A is strong enough to intercept the communication systems, targeting is to launch some integrity I attacks ${\left(A_{I}\right)}_{i}$, where i represents the possible number of integrity attacks, such as man-in-the-middle attacks and packet injection, using some solutions or built-in tools [43,51]. Thus, the contents of $T_{W}$ are maybe modified if the adversary $A_{I}$ has enough computing power to compute the hash value equivalent to the original computed value as $H_{W}$, or $H_{W}$ = $H_{A_{I}}$. Contrary, if hash values match, $H_{W}=H_{R}$, it means that adversary $A_{I}$ was not succeeded to alter $T_{W}$. However, we can examine the effectiveness of our security approach and the power of the adversary $A_{I}$, by estimating $P{\left(A_{I}\right)}_{i}$ of success and failure depending on the value of i.

### 5.4. Statement 4: IoT-BC Authentication

Authentication A can be attained for each transaction $T_{\left(W,R\right)}$ in a set $S_{3}$, if there exists a unique shared secret key $k_{\left(E,D\right)}$ and checksum CK to compute and ensure MAC (message authentication code) $M_{CK}$, and satisfy the requirements of the communication system. The condition $M_{CK}(T_{W})$ = $M_{CK}\left(T_{R}\right)$ is true, if computed MAC values are the same, and the probability P to intercept the $M_{CK}(T_{W})$ by adversary $A_{A}$ is approximately zero, $P\left(A_{A}\right)$ = 0, for each $T_{\left(W,R\right)}$.

To deploy and examine an authentication A security service in the IoT-SC system, by considering case 5 and case 6, we use a message authentication code (MAC), where the symmetric cryptography algorithm, to compute the checksum CK of each transaction $T_{\left(W,R\right)}$ in a set $S_{3}$, i.e., $S_{3}=\left\{{\left(T_{\left(R_{\left(r\right)},\;W_{\left(w\right)}\right)}\right)}_{t,t=t-1}\right\}$. A unique secret key k is generated and shared using a secure channel, meaning that key k is assumed to be secured for each transaction $T_{\left(W,R\right)}$. Let us consider case 5: edge to CS communication, we assume that ${\left(edge\right)}_{1}$, ${\left(edge\right)}_{1}\in S_{1}$, is writing a transaction $T_{W}$ to the IoT-BC controller CS, to compute the MAC value or an encrypted checksums, $M_{CK}$ is measured on $T_{W}$, such that ${\left(edge\right)}_{1}:\;M_{CK}=Comp\left(T_{W},k\right)$. Hash and MAC algorithms are relatively the same in their operations to compute arbitrary messages to some fixed size message, the only difference between them is that the MAC algorithm uses a key to generate compressed output. We suppose that $T_{W}$ is transmitted from ${\left(node\right)}_{1}$ in clear, our target is to perform authentication, not confidentiality which requires encryption function of $T_{W}$. So, during transmission, computed $M_{CK}$ is sent along with the original $T_{W}$ to CS. Upon receiving, CS uses an original $T_{W}$ received and a shared key k to compute $M_{CK}$, such that $CS\;:\;M_{CK}=Comp\left(T_{W},k\right)$. So, if computed $M_{CK}$ of CS matches to $M_{CK}$ received from ${\left(node\right)}_{1}$, i.e., $CS:\;M_{CK}=Comp\left(T_{W},k\right)$ = ${\left(node\right)}_{1}:\;M_{CK}=Comp\left(T_{W},k\right)$, we conclude that the authenticity of $T_{W}$ has been transmitted from an authorized node or ${\left(edge\right)}_{1}$. Similarly, we can compute case 6: edge to edge communication.

In the case of an adversary $A_{A}$, the IoT-SC controller CK can ensure the authenticity if the computed MAC value is not matched. However, in some cases, there is a probability P that $T_{W}$ has not been sent from the originator ${\left(node\right)}_{1}$, or the basis on the adversary $A_{A}$ computation power, and employing some intuitive attacks tools [43,51], an adversary $A_{A}$ pretends himself as the originator of $T_{W}$, but indeed it is not an authorized node. Therefore, we can demonstrate potentials or any harmful effects of an adversary by examining the $P{\left(A_{A}\right)}_{a}\left(success\right)$ and $P{\left(A_{A}\right)}_{a}\left(fail\right)$, depending on the possible number of attacks ${\left(A_{A}\right)}_{a}$, where a represents the possible number of authentication attacks to the system. The MAC algorithm, as a part of the symmetric cryptography, is efficient in its computation to provide authenticity for our defined cases.

### 5.5. Statement 5: IoT-BC Non-Repudiation

Unbreakable, non-repudiation security R can be achieved for each transaction $T_{\left(W,R\right)}$ in a set $S_{3}$, if there exists a digital signature S computing on a key pair $k_{E}(k_{pu},\;k_{pr})$, another key pair $k_{D}(k_{pu},\;k_{pr})$, a fixed-length hash value H, and satisfies the requirements of the communication system. Digital signature S can satisfy non-repudiation security R, so the probability P of interception by an adversary $A_{A}$ is exactly zero, $P\left(A_{R}\right)$ = 0, for each $T_{\left(W,R\right)}$.

For the IoT-SC system, considering case 5 and case 6, a requirement to implement and examine a non-repudiation security service is crucial, as probability P of repudiation R, i.e., $P{\left(A_{R}\right)}_{r}$, where r represents the possible number of non-repudiation attacks, an adversary $A_{R}$ acts to deny $T_{W}$ is assumed to be high. The IoT-SC system can use the public-key based digital signature scheme to effectively verify the non-repudiation security R over exchanging of each transaction $T_{W}$. We consider case 2 to compute a digital signature at the originator, we randomly selected the originator as ${\left(node\right)}_{3}$, ${\left(node\right)}_{3},\;\in S_{1}$, which uses a key pair $k_{E}(k_{pu},\;k_{pr})$, such that ${\left(node\right)}_{3}:$ $k_{E}(k_{pu},\;k_{pr})$, to perform the encryption operation employing RSA public-key cryptography algorithm and at the receiver side, SC uses another key pair $k_{D}(k_{pu},\;k_{pr})$, such that $SC:k_{D}(k_{pu},\;k_{pr})$, to perform decryption operation employing the same RSA algorithm. We generated key pairs, such as $k_{E}(k_{pu},\;k_{pr})$ and $k_{D}(k_{pu},\;k_{pr})$, locally through randomization, without the needs of a certificate authority (CA), and keys are distributed locally without the use of key distributive centers (KDC). In our study, public key $k_{pu}$ of each node in a set $S_{2}$ is known to SC; public key $k_{pu}$ is a universal address of each node in the IoT-SC system. Thus, communication between the selective originator node or ${\left(node\right)}_{3}$ and SC, public keys are known in advanced before initiate communication, and privates keys as the name suggested, these are kept private and only know to ${\left(node\right)}_{3}$ and SC. For signing $T_{W}$, ${\left(node\right)}_{3}$ first computes a hash value $T_{W}$, and later encrypts the resulted hash value using its private key $k_{pr}$, the output will be a signature signed from ${\left(node\right)}_{3}$. Thus, a signature appends with original $T_{W}$ is sent over an unsecured channel, and later, verified by SC. Upon receipt, SC uses $T_{W}$ received and inputs to the hash algorithm, i.e., SHA- 256 algorithms, to compute the output hash value. At the same time, the signature received is decrypted by the originator public key known to SC, this is actually a verification process done by the RSA algorithm, and output as hash value is then further compared with hash values computed on the original $T_{W}$ received from ${\left(node\right)}_{3}$. If both hash values verified and match, we can surely conclude the originality of the originator (or ${\left(node\right)}_{3})$. This also verifies that the originator could not repudiate signing $T_{W}$ and will not be repudiated signing future transactions. In reality, this is impossible that an adversary interception can effect non-repudiation security of IoT-SC, as a signature is created using the private key of the originator, so no one else knows its private key to perform verification; however, we imagine that this is possible in some cases when an adversary is unbelievably strong enough to act as a repudiator entity to the IoT-SC system. Therefore, we can demonstrate the harmful effects of an adversary by conducting some useful experimentations and examining the $P{\left(A_{R}\right)}_{r}\;\left(success\right)$ and $P{\left(A_{R}\right)}_{r}\;\left(fail\right)$, depending on the value of r, to the IoT-SC system. In similar manners, we can compute a digital signature by considering case 6: edge to edge communication.

### 5.6. Limitations and Enhancements

#### 5.6.1. Identity

IoT comes with several inherent concerns of privacy, as IoT interconnectivity with no-proprietary hardware, devices and sensors, usage of various software, and communication protocols provides several vulnerabilities to leaking sensitive personal information of authorized users, e.g., personal information without any usage of encryption and cryptography mechanisms. In addition, the IoT platform is not much efficient to process a massive amount of data having user privacy concerns in mind [19].

In an actual IoT platform where numerous objects are interconnected to exchange information, protecting each node identity is a great challenge, for example, commonly IoT sensors or devices exchange information, with some of their personal information, without consideration of privacy countermeasures [52]. Among other solutions, the IoT-SC system can protect each node identity by hiding a real identity, for example, a real identity can replace as a pseudonym, such as in a form of random text or a fixed size code generated using a hashing algorithm, to exchange information. Whereas in some cases, when the authenticity of the nodes is required and nodes are communicating by their pseudonyms identities or by hiding their real identities, therefore, it could be a challenge to authenticate the originator of the message. To solve this issue, two solutions: (1) Pseudonym certification authority (PCA), is authorized to establish and issue pseudonym certificates, depending on anonymized credentials, to devices or nodes; (2) Anonymous authentication system, uses cryptography and digital signature scheme to enhance the user privacy and provides a solution to authenticate anonymity of the user: an anonymous user participating in the system [53,54,55,56]. IoT can use pseudonym certification authority (PCA) to attain the privacy of its objects during communication—a temporary credential, or one-time use anonymized credential, can be used as a pseudonym identity of a node to the system, or for a node that shows interest to join the network to exchange information. It is important that node anonymous credentials can be cryptographically proven before participating in the network. Therefore, by doing so, each node identity will not be disclosed to others, e.g., to unauthorized entities, adversaries, or third parties, and the IoT system can ensure node authenticity. Anonymized credential as pseudonyms should used once, therefore, to avoid any adversarial activities being linked to the system. Moreover, in a situation when nodes are connected as a standalone entity or nodes are associated to a single network group, for example, smart home application, it is also possible that each can participant anonymously in the system. Authentication through cryptography approaches, a node in a network can revoke the session key if there is another node behavior-changing exception. By doing this, the node can keep its privacy by not disclosing its identity to others.

#### 5.6.2. Location

In IoT, even in this study, location is a position of objects, e.g., edge nodes, device/sensors, or person, geographical positions. IoT and its applications have been extensively employed for tracking and monitoring of various objects remotely in real time. In the IoT-SC system, specifically for the cold food supply, real-time monitoring of information using sensors is crucial for the effective end-to-end supply chain. Tacking of products in warehouses and during transportation are important concerns and can be accomplished efficiently through IoT: tracking and monitoring of goods remotely. However, in the IoT-SC system, location access services open new privacy challenges, or threats, such as localization, profiling, and identification, to reveal the personal sensitive information of users, devices, and sensors distributed across several locations and connecting over the internet. To protect location privacy, several solutions have been conducted in [24,56,57,58], which can be employed in IoT case studies, e.g., the IoT-SC system. Cloaking, a location anonymity mechanism, employs to hide a person’s original location to protect location privacy [25]; location obfuscation, the mechanism, such as pseudonyms, rounding, or spatial/invisible cloaking, employs in location-based services to protect location privacy through changing the actual location of the user [24]; dummy-based mechanism, protects user location privacy by allowing the user to send numerous dummy position coordinates instead of the actual location, therefore an adversary cannot link to the actual location of the user [57]; false-based location mechanism, allowing the user to send false or fake location information instead of reallocation, to protect the location privacy that can be leaked by the adversary [58].

#### 5.6.3. Confidentiality

In the situation of IoT, e.g., the IoT-SC system, numerous devices and sensors are installed and networked to pre-processed data periodically to the IoT server for storage and further analysis. Therefore, collecting information from IoT objects is sensitive and important to keep secure, not to be leaked maliciously; ensuring the confidentiality of information is important for IoT. In literature [3,6,43,51], numerous solutions, especially cryptography algorithms as strong security solutions, are used to enhance various security challenges of IoT; cryptography encryption operations are significant to protect IoT sensitive information from adversaries. However, for IoT, a network composes a massive number of objects, it is difficult to protect the confidentiality of information from millions of objects, even with edge/fog connectivity, employing cryptography encryption algorithms [51,59]. Among others, the IoT central platform is not able to generate and manage a large number of keys for millions of transactions; further, the IoT platform relies on thirty party certificate author (CA) for digital certification and key distribution center (KDC) for keys exchanging and minimizing their risks. Similarly, for the IoT-SC system, the information generates and collects, having a number of keys, from a large number of objects, both asymmetric and asymmetric cryptography mechanisms used to attain information confidentiality, is challenging.

#### 5.6.4. Authentication

Like traditional systems, IoT indeed must have a robust authentication mechanism(s): the IoT-SC system acknowledges that information received from nodes is authentic, or participant nodes are real or are authentic users. In cases of the potential adversary impersonating as an authorized user to the IoT-SC system, it is critical to authentic information or the sender that the user is communicating with other valid entities, not with adversaries. Therefore, this is important for the IoT-SC system to share information with authentic nodes only, if IoT-SC is not sure about the objects its connecting with, IoT-SC enables the protection of its sensitive information that is shared or received from adversaries. Numerous security solutions, including authentication protocols, have been deployed to authenticate information and users as valid entities [22,59]. Mainly the solutions are concerned to authenticate a user using passwords to login to the system, employing transport layer security (TLS)/Secure Sockets Layer (SSL) protocol, tokenization, and public-key cryptography. These solutions and their developments are good enough for several applications, and systems including web browsers, and are commonly used by IoT. However, the evolution of advanced technologies and available computing powers, like conventional communication systems, IoT is also suffering from vulnerabilities and potentials authenticate attacks, therefore, to provide authentication for an IoT-scale platform is relatively more challenging than traditional systems [6,40].

#### 5.6.5. Non-Repudiation

For the IoT-SC system, the MAC algorithm, or other symmetric algorithms including hashing, is not able to provide non-repudiation security service, among others [40,51]. To achieve effectiveness in the IoT-SC communication system, a non-repudiation security service, confirmation, or guarantee that any node in a set cannot repudiate or contradict any prior transmitted transaction and this security service is very useful for a complex and scaled IoT network. Alone, symmetric encryption, asymmetric encryption, or hashing are not much efficient to provide the non-repudiation security service to the IoT-SC system, for example, if there is a dispute among nodes over origination, no one indeed can prove the who is a real originator of the transaction. Specifically, in the case of IoT massive connectivity, where nodes autonomously exchange information, it is almost impossible that the IoT-centric computing server leverages a non-repudiation security service among massively connected nodes. For example, if the originator node continually opposes transactions and states that the receiving node already copied transaction fraudulently, therefore, in this critical situation, it is not possible to decide who is a real originator of the transaction or who is acting fraudulently. Therefore, to provide a non-repudiation security service where an originator does not contradict the transaction, public cryptography digital signature schemes can be solutions to these issues [60]. Employing a digital signature scheme, a real identity of the originator is bind with a transaction that a sender indents to transmit, and later will be validated independently at the receiving side or in the case of third-party verification.

## 6. Results and Discussion

The IoT-SC system and its components, such as nodes, edge nodes, and a controller, are programmed using visual studio C#, i.e., net core is used to program a complete IoT-SC system, and Azure SQL data or Azure cloud is used to record every transaction. In the IoT-SC system, IoT nodes, such as IoT-Enabled RFID active readers, are assumed to be connected through Raspberry Pi RFID RC522 and is connected to C# GUI. In the IoT-SC system, eight main commands are used to perform system operations or transactions, and are detailed in Table 2.

**Figure 4:**
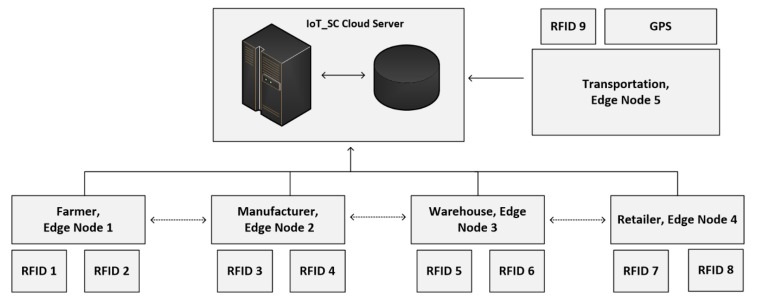
A setup and nodes connectivity of the IoT-SC system: each edge node is responsible to carry specific operations through its connected nodes, operations are a number of transactions written to IoT-SC controller: edge nodes are only authorized to write transactions to IoT-SC controller. To provide an end-to-end supply chain, each edge node performs the desire operations time-to-time and writes as transactions to the IoT-SC controller.

To conduct the experimentation and performance results, we limit the number of nodes and edge nodes in sets $S_{1}$ and $S_{2}$, such that, $S_{1}=\left\{{\left(node\right)}_{\;n=10}\right\}.=\left\{{\left(node\right)}_{0},{\left(node\right)}_{1},{\left(node\right)}_{2},\ldots \ldots ,\;{\left(node\right)}_{9}\right\}$ and $S_{2}=\left\{\left(\mathrm{edge}{)}_{e=5}\right)\right\}.=\left\{{\left(edge\right)}_{0},{\left(edge\right)}_{1},{\left(edge\right)}_{2},{\left(edge\right)}_{3},\;{\left(edge\right)}_{4}\right\}$. Numbers of edge nodes in $S_{2}$, where e=5, are designated to carry the operations of supply chain entities: farmer, manufacturer, warehouse, retailer, and transporter. Here, we do not use the customer entity because this entity can only view the product information and is not connected to the IoT-SC system. Therefore, to perform the operations of each supply chain entity, the total are five entities, we designate ${\left(edge\right)}_{0}$ to farmer, ${\left(edge\right)}_{1}$ to manufacturer, ${\left(edge\right)}_{2}$ to the warehouse, ${\left(edge\right)}_{3}$ to retailer, and ${\left(edge\right)}_{4}$ to transporter. Means that, edge nodes in a set $S_{2}$, $S_{2}=\left\{\left(\mathrm{edge}{)}_{e=5}\right)\right\}$, are designed to carry the operations of supply chain entities, for example, each edge node reads transactions from IoT-enabled RFID readers, and recorder onto its local storage. We assumed that ${\left(edge\right)}_{0}$ to ${\left(edge\right)}_{3}$ are distributed across various locations, with unknown location coordinates, and are linked to the IoT-SC controller via the internet. However, ${\left(edge\right)}_{4}$ is a moving entity, so its location coordinates changing time-to-time while carrying transportations. Each edge node in a set $S_{2}$ is connected to two IoT-enabled RFID active readers, to carry supply chain operations. Operations are the number of transactions carried by each edge node and then, written to the IoT-SC controller, as edges are only authorized to write transactions, not to read. However, supply chain entities or users can view or read transactions, for example, to trace record history by requesting or/and accessing the IoT-SC controller. In a set $S_{2}$, ${\left(edge\right)}_{0}$ to ${\left(edge\right)}_{3}$ are connected to two IoT objects: IoT-enabled RFID active readers; ${\left(edge\right)}_{4}$ is connected to an IoT-enabled RFID active reader and an IoT-enabled GPS device, it is useful as ${\left(edge\right)}_{4}$ geographical location changes time-to-time, for example, during transportations between supply chain entities; we assumed that geographical locations of ${\left(edge\right)}_{0}$ to ${\left(edge\right)}_{3}$ are fixed, and their individual fixed positional coordinates (longitude and attitude) are unknown and recorded on the IoT-SC controller. Meaning that, each edge node works as an intermediary node between nodes and IoT-SC controller, and has small memory storage, enough computation power for processing information, and a reliable internet connection; unfortunately, analytical capabilities are not available locally in edge nodes, however, IoT-SC controller provides analytical capabilities to process information and takes corresponding actions accordingly. We assume that nodes in a set $S_{1}$ are representing nine IoT-enabled RFID active readers, and one IoT-enabled GPS device, installed and networked in the IoT-SC system. Such that, ${\left(node\right)}_{0}$ and ${\left(node\right)}_{1}$ are represented as IoT-enabled RFID active readers, and connected to ${\left(edge\right)}_{0}$, $\left\{{\left(node\right)}_{0},{\left(node\right)}_{1}\right\}\in \;{\left(edge\right)}_{0}$; similarly, we can assume that, $\left\{{\left(node\right)}_{2},{\left(node\right)}_{3}\right\}\in \;{\left(edge\right)}_{1}$, $\left\{{\left(node\right)}_{4},{\left(node\right)}_{5}\right\}\in \;{\left(edge\right)}_{2}$, $\left\{{\left(node\right)}_{6},{\left(node\right)}_{7}\right\}\in \;{\left(edge\right)}_{3}$, and $\left\{{\left(node\right)}_{8},{\left(node\right)}_{9}\right\}\in \;{\left(edge\right)}_{4}$, ${\left(node\right)}_{9}$ is an IoT-enabled GPS device. Figure 4 illustrates a setup and nodes connectivity of the IoT-SC system.

As mentioned, edge nodes carry the specific operations of supply chain entities: farmer, manufacturer, warehouse, retailer, and transporter; however, the details of operations performed by supply chain entities are not under the scope of this study; at the present, we are only interested in collecting and processing information from nodes to edge nodes to the IoT-SC controller, or depending on the defined communication cases, which are:

- At the farmer side, ${\left(edge\right)}_{0}$ reads information from ${\left(node\right)}_{0}$ and ${\left(node\right)}_{1}$, and then writes to the IoT-SC controller. ${\left(node\right)}_{0}$ collects information via active RFID tag fixed to raw material. We assume that raw material, a quantity of 100 Kgs, is stored in a big cotton bag, which means each bag can store 100 Kgs of weight and is tagged to prove its identity in the supply chain.
- At the manufacturer, ${\left(edge\right)}_{1}$ reads information from ${\left(node\right)}_{2}$ and ${\left(node\right)}_{3}$, and then writes to the IoT-SC controller. ${\left(node\right)}_{2}$ collects information via active RFID tag fixed to packets. We assume that raw material assembles to fixed-size packets, each packet size is only 1 Kg, and packed to a cotton box. Each cotton box can contain 10 packets. Each packet is tagged, including labeling the detail of manufacture date, expiry date, and ingredients.
- At the warehouse side, ${\left(edge\right)}_{2}$ reads information from ${\left(node\right)}_{4}$ and ${\left(node\right)}_{5},$ and then writes to the IoT-SC controller. Similarly, ${\left(node\right)}_{4}$ collects information via active RFID tag fixed to cotton boxes or pallets. In general, the warehouse is responsible to manage an effectivity inventory. Inventory management is a complex process in a medium and large size warehouse. IoT is an ultimate solution for warehouse and inventory management, however, these features are not under the scope of this study. For instance, we are interested to read information from ${\left(node\right)}_{4}$ and ${\left(node\right)}_{5}$ via ${\left(edge\right)}_{2}$ will further process to the IoT-SC controller.
- At the retailer side, ${\left(edge\right)}_{3}$ reads information from ${\left(node\right)}_{6}$ and ${\left(node\right)}_{7},$ and then writes to the IoT-SC controller. For example, the retailer opens the boxes or pallets received from the warehouse, read tags information, and writes to the IoT-SC controller via ${\left(edge\right)}_{3}$.
- The transportation entity is common among all other entities, as its performance transports between entities. In our case, transportation is installed with ${\left(node\right)}_{8}$ and ${\left(node\right)}_{9}$. Every time, material or boxes, or pallets are loaded/unloaded to/from transportation in a supply chain, ${\left(edge\right)}_{4}$ reads information from ${\left(node\right)}_{8}$, with information of location coordinates via ${\left(node\right)}_{9}$, and uploads to the IoT-SC controller.

To examine the effectiveness of the proposed implementation, this study defines communication rules and cases to measure and evaluate the performance results in the IoT-SC system. For that, six communication cases, having specific rules, are defined to test and examine the performances of proposed privacy and security statements. In total, there are five statements: statement 1 is a privacy statement, and statements 2 to 5 are the security statements. For simplicity, four communications cases, i.e., case 1 to 4, define and consider in turn to test and examine privacy statement, and the remaining 2 communication cases, i.e., case 5 and 6, are defined and considered in turn to test and examine security statements: Statement 1: Confidentiality, Statement 2: Integrity, Statement 3: Authentication, and Statement 4: Non-repudiation. Note that, for all communications cases, i.e., case 1 to 6, the IoT-SC controller is superior, as it registers all the nodes and edge nodes, sets communication rules for each defined case, and can alter the rules based on the communication requirements. The IoT-SC controller uses each node/edge node real identity, e.g., node ID or address, node type, node model, manufacture ID, and firmware version, and location coordinates, as each node/edge node information is known and recorded on the IoT-SC controller, to generate a fixed-size hash code that is used as a pseudonym to hide each node/edge node real identity. SC is responsible to perform these tasks, including to write new pseudonyms to each node firmware, or to update the firmware with a new hash code. Communication cases, i.e., cases 1 to 6, are detailed as follows:

- Case 1: nodes to edge communication—Nodes are the actual IoT objects installed and networked to get measurements, for example, to read RFID active tag information and write to edge nodes continuously. For privacy measurements, P(I, L), we allocate two nodes to an edge node, i.e., $\left\{{\left(node\right)}_{0},{\left(node\right)}_{1}\right\}\in \;{\left(edge\right)}_{0}$ and so on, meaning that only ${\left(node\right)}_{0}$ and ${\left(node\right)}_{1}$ are allowed writing transaction $T_{W}$ to ${\left(edge\right)}_{0}$. In open network connectivity, for each transaction $T_{W}$, every node in a set $S_{1}$, $S_{1}=\left\{{\left(node\right)}_{\;n=10}\right\}$ conceals its real identity and location, through initiating communication with a pseudonym to edge nodes in a set $S_{2}$, $S_{2}=\left\{\left(\mathrm{edge}{)}_{e=5}\right)\right\}$. A pseudonym is a unique hash code the represents a node’s identity, including its location, instead of its real identity. As mentioned, the IoT-SC controller always keeps a record of nodes and edge nodes, and their unique hash codes as a pseudonym. For communication case 1, each edge node keeps a replicate copy of the hash codes of its belonging nodes; in other words, the IoT-SC controller writes the hash codes of selective nodes to a specific edge node. So, during communication, nodes use their pseudonyms to hide their actual identities, and on the other side, a specific edge node can verify the identities of the nodes, as authorized identities to the system, by matching their hash codes recorded onto the edge node.
- Case 2: nodes to SC communication—This communication case is unusual and occurs when every edge node in a set $S_{2}$, $S_{2}=\left\{\left(\mathrm{edge}{)}_{e=5}\right)\right\}$ is offline continuously or is not able to verify the originator, thus, a node in a set $S_{1}$, $S_{1}=\left\{{\left(node\right)}_{\;n=10}\right\}$ can use its pseudonym to initiate and write the transaction $T_{W}$ to the IoT-SC controller. The IoT-SC controller verifies the originator of the transaction $T_{W}$ by matching the hash code.
- Case 3: edge to edge communication—In the IoT-SC system, edge nodes in a set $S_{2}$, $S_{2}=\left\{\left(\mathrm{edge}{)}_{e=5}\right)\right\}$ are not known to each other, thus an edge node, e.g., ${\left(edge\right)}_{0}$, can use its pseudonym to initiate communication to another edge node, e.g., ${\left(edge\right)}_{1}$. In this case, ${\left(edge\right)}_{1}$ can only verify the originator via the IoT-SC controller. To conduct the privacy measurements, P(I, L), we assume that each edge node in a set $S_{2}$, $S_{2}=\left\{\left(\mathrm{edge}{)}_{e=5}\right)\right\}$ keeps a copy of the hash codes of other edge nodes.
- Case 4: edge to CS communication—Like case 1, each edge node in a set $S_{2}$, $S_{2}=\left\{\left(\mathrm{edge}{)}_{e=5}\right)\right\}$ can use its pseudonym to initiate and write the transaction $T_{W}$ to the IoT-SC controller. The IoT-SC controller verifies the originator of a transaction $T_{W}$ by matching the hash code.
- Case 5: edge to CS communication—In general, edge nodes are efficient in computation power, increasing response time, reducing bandwidth, and may have analytical capabilities. However, in our case, these nodes are limited in their computation power, storage, and have null analytical capabilities; due to these limitations, each transaction $T_{W}$, for example, after a while or after 1–2 h, recorded on edge will be shifted to the IoT-SC controller, for permanent storage and further analytical purposes. There is some probability that storage will be lost because of some obstacles and hardware errors. So, while transferring information in an open network, edge nodes can exchange information securely by deploying and examining security statements 2 to 5.
- Case 6: edge and edge communication—This communication case is critical and occurs when one edge node may behave abnormally or in a situation with an edge node being offline continuously due to some issues. For example, ${\left(edge\right)}_{1}$ has been reading transaction $T_{R}$ from ${\left(node\right)}_{2}$ and ${\left(node\right)}_{3}$, i.e., $\left\{{\left(node\right)}_{2},{\left(node\right)}_{3}\right\}\in \;{\left(edge\right)}_{1}$, and after a time period, writing transaction $T_{W}$ to IoT-SC controller, continuously. Over a time period, ${\left(edge\right)}_{1}$ stopped writing transaction $T_{W}$, or in situation IoT-SC control requests for transaction $T_{R}$ but ${\left(edge\right)}_{1}$ status is offline continuously. Therefore, to provide reliable communication, the IoT-SC controller redirects communication from ${\left(edge\right)}_{1}$ to other closer edge node; means that, nodes connected to ${\left(edge\right)}_{1}$ are redirected or allocated to another edge node, e.g., ${\left(edge\right)}_{2}$. Nodes, i.e., $\left\{{\left(node\right)}_{2},{\left(node\right)}_{3}\right\}$, are also updated with new edge node or ${\left(edge\right)}_{2}$ configuration if there are any. However, there may be an effect on the ${\left(edge\right)}_{2}$ throughput if it has been already overloaded with transactions from ${\left(node\right)}_{4}$ and ${\left(node\right)}_{5}.$ So, while transferring information in an open network, security statements 2 to 5 are deployed to exchange information securely to the IoT-SC controller.

In general, this is not possible for IoT objects to be installed with security mechanisms like cryptography mechanisms, which require enough computational power and complex operations to attain security, specifically for encryption and decryption operations. However, lightweight cryptography and other security mechanisms can be solutions to this problem to install security solutions as parts of smart objects [5,40]. In this study, we consider a similar approach where nodes connected to the system are not installed with any security solution or any security service 2 to 5, but these nodes can execute privacy statements as a solution to hide their real identities, for that, hash codes are used.

**Figure 5:**
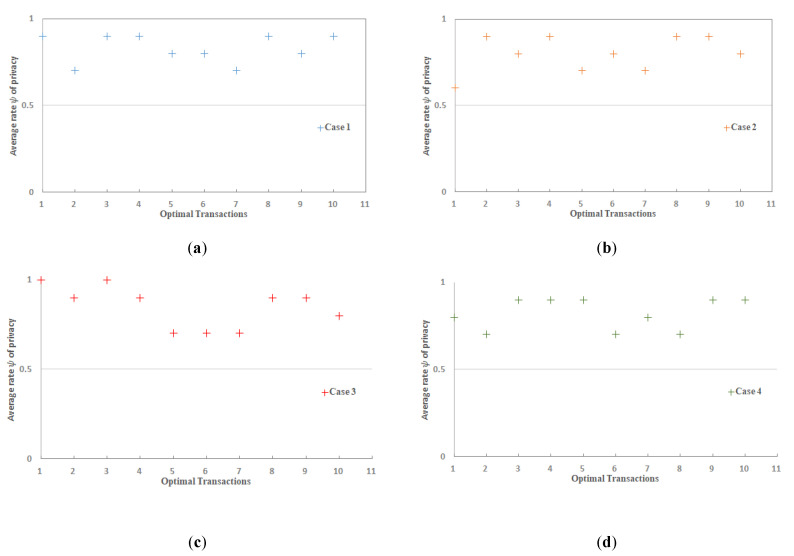
(**a**) Average rate ψ of privacy for Case 1; (**b**) Average rate ψ of privacy for Case 2; (**c**) Average rate ψ of privacy for Case 3; (**d**) Average rate ψ of privacy for Case 4.

To test and examine the privacy, considering communication cases 1 to 4, a number of experiments are conducted to measure the performance results. Among other several experiments, optimal successful experiments are selected, their measurements, the throughputs as rates of privacy P(I, L) are carefully observed, and are illustrated in Figure 4. During experimentation, we assume that the whole IoT-SC network is up, there are no network issues and other communication obstacles. For that, we conducted an experiment 0 illustrated in Figure 4, which demonstrates that the IoT-SC network is working well and up for communication cases 1 to 4. Considering communication cases, we transmit a null payload for each successful experiment, as our intention is to verify the privacy of each valid node/edge node and to measure the rate ψ of privacy P(I, L), i.e., high or low, at the received end, for examples: (1) considering communication case 1, we transmitted a null payload a number of times from randomly selective nodes, using pseudonyms instead of the real identities, and measured by the rate ψ of privacy P(I, L) at the IoT-SC controller; (2) Considering communication case 2 and 3, edge nodes will be measured by the rate ψ of privacy P(I, L), depending upon payload (null) successfully received from nodes, and their hashes verified, however communication case 3 may vary in the situations; (3) Similarly, considering communication case 4, we transmit a null payload a number of times from randomly selective edge nodes, using pseudonyms instead of their real identities, and measured the rate ψ of privacy P(I, L) at the IoT-SC controller. Meaning that, the rate ψ of privacy P(I, L) is either low or high, high rate ψ(high) can be computed if the receiver-end received a payload (null) from a transmitter, considering cases 1 to 4, and its pseudonym matches to the hash code recorded on the receiver end, otherwise, the rate will be low ψ(low) as hash codes are not verifiable or not valid hash codes. As parts of Figure 5: Figure 5a demonstrates the optimal successful experiments and throughputs, rate ψ of privacy P(I, L), considering commination case 1; similarly, Figure 5b–d demonstrates the optimal successfully experiments and throughputs, considering commination cases 2, 3, and 4. For a better representation of ψ(high) and ψ(low), we assume that 0.5 is a calculated average rate μ, a lower bound value φ is set on 0.4, and an upper bound value ϕ is set on μ = 0.5; therefore, rate ψ of privacy P(I, L) is assumed to be high or ψ(high), if probability P of each transaction $T_{W}$ in a set $S_{3}$ lies between 0.5 and 1.0, otherwise considered as ψ(low).

**Figure 6:**
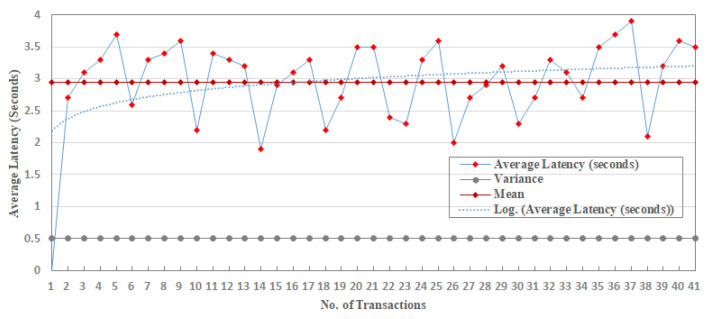
Average latency considering communication cases 5 and 6.

**Figure 7:**
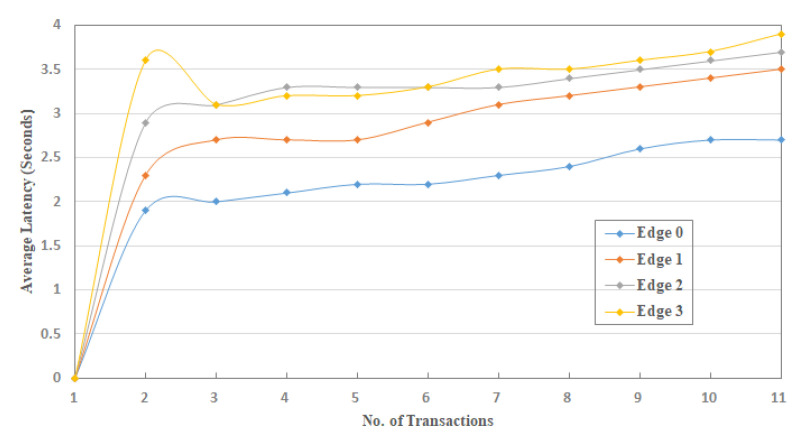
Average latency computed by edge nodes.

**Figure 8:**
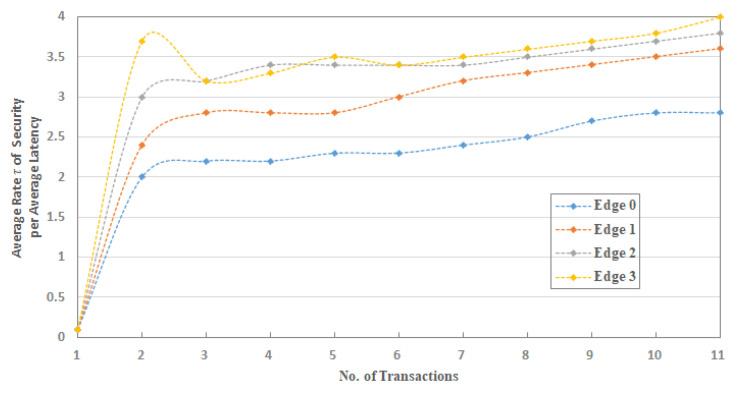
Average rate τ of security.

**Figure 9:**
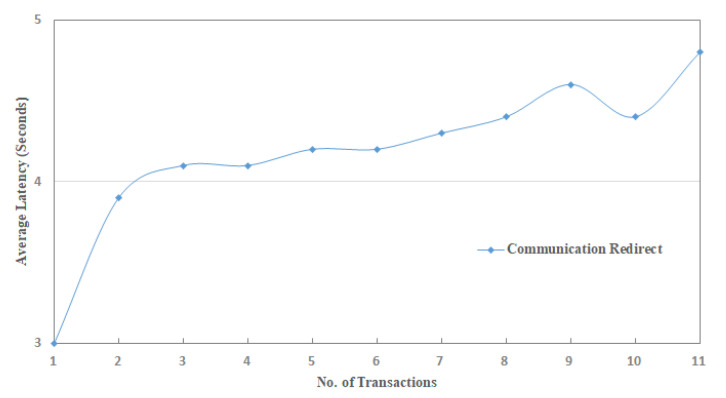
Average latency for communication redirect.

For security measurements, average optimal numbers of successful experiments are selected, and throughputs: latency and rate of security τ, are carefully observed, considering communication cases 5 and 6. Latency is the time interval when each transaction $T_{W}$ writes from edge node and successfully received or read by IoT-SC controller and similarly rate of security τ is computed when IoT-SC successfully verifies the security, i.e., confidentiality C, integrity H, authentication A, and non-repudiation R, of each write transaction $T_{W}$ from edge nodes. We merge communication cases 5 and 6 or consider these cases as one communication case, because in both communication cases, each transaction writes from edge nodes to the IoT-SC controller. We assume that a number of transactions from nodes are successfully carried out, without any issues of network connectivity, communication obstacles, and are recorded on edge nodes, i.e., $\left\{{\left(node\right)}_{0},{\left(node\right)}_{1}\right\}\in \;{\left(edge\right)}_{0},$ and so on. Figure 6 exhibits the average latency of a selective number of transactions done, considering communication cases 5 and 6. Figure 7 illustrates security services, such as confidentiality, integrity, authentication, and non-repudiations, are tested to measure the latency, and Figure 8 illustrates to examine the rate of security τ based on Figure 7 performance results, form edge nodes to the IoT-SC controller. The performance results in Figure 6, Figure 7 and Figure 8 are measured in absences of network issues and any communication obstacles, and each edge node in a set $S_{2}$, $S_{2}=\left\{\left(\mathrm{edge}{)}_{e=5}\right)\right\}$ is randomly selected to write a transaction $T_{W}$ in turn to the IoT-SC controller, not in a batch. Figure 9 shows the average latency or time an IoT-SC controller requires to redirect the communication from one edge node to other; however, average latency will be increased as increasing a number of nodes to edge nodes; in other words, the IoT-SC controller requires more time to redirect the communication if there are a number of nodes connected to the offline edge node.

Forming the given proofs of privacy and security statements and examining of privacy and security rates, we conclude that probability of adversaries: the probability of the attacker on privacy $P{\left(A_{P}\right)}_{p}$, probability of the attacker on confidentiality $P{\left(A_{C}\right)}_{c}$, probability of the attacker on integrity $P{\left(A_{I}\right)}_{i}$, probability of the attacker on authentication $P{\left(A_{A}\right)}_{a}$, probability of the attacker on non-repudiation $P{\left(A_{R}\right)}_{r}$, are assumed to be very low, or approximately equal to zero. Table 3 depicts the results of validation against the probability of adversaries.

## 7. Conclusions

Supply chain management (SCM) systems have been playing important roles and have great importance for an enormous number of industries and organizations, however, to fulfill the complex operational requirements and massively growing demands of the supply chain, therefore, there are several challenges, mainly scalability, privacy, and security. It is obvious, as the demands increased, SCM systems must be further scaled and robust to combat current and prospective operational and as well as communication challenges. Powering IoT technology for SCM systems can feature to manage supply chain overall operations more efficiently, track and monitoring goods, traceability, and analytical capabilities to take some useful decisions, in real time. Indeed, as we examined in this study, providing and managing privacy and information security in massively dense networks are the main challenges for the IoT centralized platform. To combat this, we modeled a scaled IoT-based supply chain system and examined thoroughly from the existing literature the main privacy and security challenges as parts of IoT-SC, or IoT interconnectivity with SCM. Therefore, we proposed and deployed solutions to protect the privacy of objects (for example, edge nodes), and to address the security of each transaction by providing main security services, i.e., CIA, in the IoT-SC system. For some worst-case scenarios, i.e., potential adversary interceptions, the digital signature scheme is deployed to ensure non-repudiation security service. Further, to examine the effectiveness of the overall system, formal privacy, and security statements, including potential interceptions of adversaries, and measurements are conducted. We conclude that the proposed solutions well-addressed the selective privacy and security challenges of the IoT-SC system.

## 8. Future Works

Over the years, IoT and blockchain technology demands have been growing rapidly for various applications and systems, including industries and manufacturing [40], therefore, there is great interest to use these two emerging technologies as an integrated platform for several use cases [40,61]. Combining IoT and blockchain technologies can revolutionize the world to get the benefits of vast objects’ connectivity and features to process and record a large amount of data with more efficiency, privacy, and security. Blockchain uses with IoT, IoT can overcome its several inherent main issues, for example, it can record all its transactions using decentralized technology, with more supports of immutability and suitability. Blockchain has interesting features of validation and smart contracts, which are very useful for IoT to manage its million of transactions in more authentic ways. Using blockchain, IoT can maintain all its information into the cloud storage and can maintain privacy and security of information [62]; cryptography hashing function, as a key component of blockchain, can provide great immutability for IoT transactions to be recorded onto blockchain, in other words, IoT can migrate its cloud-storage to the blockchain immutable ledger system. IoT, a massively scaled platform, requires careful intention to maintain the information security, including main security features: confidentiality, integrity and authentication, and privacy aspects [40,63]: objects identification, monitoring, and tracking locations, information mishandling, etc. For example, in the IoT-SC system, large connectivity is challenging and it is almost impossible to design and install security and privacy models for each IoT node, usually, IoT nodes have limited resources of power and computation, and at another side, IoT central systems are not able to provide security and verify privacy to largely connected nodes (i.e., SCM system).

In future works, we plan to use an integrated IoT and blockchain system to overcome these issues of security and privacy more effectively. In short, we will simply replace the IoT cloud central storage system to decentralized and distributive blockchain cloud storage, and later examine that blockchain replacement will be significant to the existing IoT-SC system. However, we are sure that blockchain collaboration with IoT will be a robust, integrated solution to further achieve end-to-end supply chain, and will be an effective solution to address the given security and privacy challenges of the IoT-SC system.

## Figures and Tables

**Table 1:** System terminologies.

No.	Terminologies	Description
1.	$S_{1}=\left\{{\left(node\right)}_{n,n=n-1}\right\};\;S_{2}=\left\{\left(\mathrm{edge}{)}_{e,e=e-1}\right)\right\}$.	$S_{1}$ representing n number of nodes; $S_{2}$ representing e number of edge nodes.
2.	$S_{3}=\left\{\left(T_{\left(R_{\left(r\right)},W_{\left(w\right)}\right)}{)}_{t,t=t-1}\right)\right\}$,	$S_{3}$ representing t number of read/write transactions $T_{\left(R,W\right)}.$
3.	$S_{4}={\left(I,\;L\right)}_{S_{1}}$; $S_{5}={\left(I,\;L\right)}_{S_{2}}$	A unique identity I and location coordinates L of each node and edge node in sets $S_{1}and\;S_{2}.$
4.	${\left(A_{P}\right)}_{p}$; $P{\left(A_{P}\right)}_{p}$	A number of adversaries on privacy; the probability of attempts.
5.	${\left(A_{C}\right)}_{c}$; ${\left(A_{I}\right)}_{i}$; $P\left(A_{A}\right)$; ${\left(A_{R}\right)}_{r}$	A number of adversaries: confidentiality; Integrity; Authentication; Non-repudiation.
6.	$P\left(A_{C}\right)$; $P{\left(A_{I}\right)}_{i}$; $P{\left(A_{A}\right)}_{a}$; $P{\left(A_{R}\right)}_{r}$	Probability of attempts: confidentiality; Integrity; Authentication; Non-repudiation.
7.	$k_{E}(k_{pu},\;k_{pr})$; $k_{D}(k_{pu},\;k_{pr})$	Key pairs for encryption and decryption.

**Table 2:** System commands.

	Commands, CMD	Descriptions
1.	T(0001, 1)	Read transaction from IoT-SC controller, with Confirmation bit.
2.	T(0001, 0)	Read transaction from IoT-SC controller, without Confirmation bit.
3.	T(0011, 1)	Write transaction to IoT-SC controller, with Confirmation bit.
4.	T(0011, 0)	Read transaction to IoT-SC controller, without Confirmation bit.
5.	T(0111, 1)	Critical-controller mode.
6.	T(1111, 1)	Critical-edge mode.
7.	T(0111, 0)	Node-shift mode.
8.	T(1111, 0)	Edge-shift mode.

**Table 3:** Validations.

$S_{1}/S_{2}$	H	$P\left(A_{p}\right)$	$P\left(A_{C}\right)$	$P\left(A_{I}\right)$	$P\left(A_{A}\right)$	$P\left(A_{R}\right)$
${\left(edge\right)}_{0}$	721aa745f323703bb4d678e6bd2fb34faa5b9d75f5b39f366d578d0210f62430	≈0	≈0	≈0	≈0	≈0
${\left(edge\right)}_{1}$	a7d066270ce19d0b0c345b40b2fa9b5ae9599e46cc5955e2bb745e6f34af71a1	≈0	≈0	≈0	≈0	≈0
${\left(edge\right)}_{2}$	b59b1856fdfd873a0ac601dc93a0200b7118c0b217543bbf0ce7ae586a6cb07e	≈0	≈0	≈0	≈0	≈0
${\left(edge\right)}_{3}$	07a0c7ae901b9527ad8df0e650d348b21ce9feeb7855219503b0f63b3f82da45	≈0	≈0	≈0	≈0	≈0
${\left(edge\right)}_{4}$	36c69cc5ed2ff5c614b83832b9774a4a4392927428be4da8a260367907d60b7f	≈0	≈0	≈0	≈0	≈0
${\left(node\right)}_{0}$	7d79b337a3afaa02cf9551c725fe9dde23137f4a403671c96cbc41d7b0515a36	≈0	≈0	≈0	≈0	≈0
${\left(node\right)}_{1}$	5eec305934c303ed15547004c0a9ca0e57d57ce181b97b5e847056ba48ff4c9e	≈0	≈0	≈0	≈0	≈0
${\left(node\right)}_{2}$	a4338d77aeaf48bfa72ebff1c1bfc73140bd4ed0e8450a230d91eca4df156677	≈0	≈0	≈0	≈0	≈0
${\left(node\right)}_{3}$	8e1c4acfcc5862be269e08b50fa442c2c2aaff595d33e82ee47cf5c999de973a	≈0	≈0	≈0	≈0	≈0
${\left(node\right)}_{4}$	ec57d117ebdc725073052ac2861542361d59bdbe00061a6d3120c835e5914482	≈0	≈0	≈0	≈0	≈0
${\left(node\right)}_{5}$	4d2570ae812a88dfd0204895b7528389e0a28e6f92d5aa04c63550dd6dd3c8cc	≈0	≈0	≈0	≈0	≈0
${\left(node\right)}_{6}$	65f5f1b1c81afc7de0184bd924e1b237d819bb4dd5c4041ab17e2e51f3e5f0cd	≈0	≈0	≈0	≈0	≈0
${\left(node\right)}_{7}$	65d6ab455e306709a3e8c26b124d2140851e318d4f9031bd022769b66aecfb6f	≈0	≈0	≈0	≈0	≈0
${\left(node\right)}_{8}$	b786c77378ad1599759959e113ee0ce2d1a9e8126f28eeb8082182c0e1387390	≈0	≈0	≈0	≈0	≈0
${\left(node\right)}_{9}$	ee487eb8e4591f3ad743706718e2dfbd977f2eecee6fa595625a6b691772642d	≈0	≈0.	≈0	≈0	≈0
